# A genome triplication associated with early diversification of the core eudicots

**DOI:** 10.1186/gb-2012-13-1-r3

**Published:** 2012-01-26

**Authors:** Yuannian Jiao, Jim Leebens-Mack, Saravanaraj Ayyampalayam, John E Bowers, Michael R McKain, Joel McNeal, Megan Rolf, Daniel R Ruzicka, Eric Wafula, Norman J Wickett, Xiaolei Wu, Yong Zhang, Jun Wang, Yeting Zhang, Eric J Carpenter, Michael K Deyholos, Toni M Kutchan, Andre S Chanderbali, Pamela S Soltis, Dennis W Stevenson, Richard McCombie, J Chris Pires, Gane Ka-Shu Wong, Douglas E Soltis, Claude W dePamphilis

**Affiliations:** 1 Intercollege Graduate Degree Program in Plant Biology, The Pennsylvania State University, University Park, PA 16802, USA; 2 Department of Biology, Institute of Molecular Evolutionary Genetics, Huck Institutes of the Life Sciences, The Pennsylvania State University, University Park, PA 16802, USA; 3 Department of Plant Biology, University of Georgia, Athens, GA 30602, USA; 4 Department of Biology and Physics, Kennesaw State University, Kennesaw, GA 30144, USA; 5 Donald Danforth Plant Science Center, 975 North Warson Road, St Louis, MO 63132, USA; 6 Division of Plant Science and Conservation, Chicago Botanic Garden, Glencoe, IL 60022, USA; 7 Beijing Genomics Institute-Shenzhen, Bei Shan Industrial Zone, Yantian District, Shenzhen 518083, China; 8 The Novo Nordisk Foundation Center for Basic Metabolic Research, Department of Biology, University of Copenhagen, Store Kannikestræde 11, 1169 København K, Denmark; 9 Intercollege Graduate Degree Program in Genetics, The Pennsylvania State University, University Park, PA 16802, USA; 10 Department of Biological Sciences, University of Alberta, Edmonton, Alberta T6G 2E9, Canada; 11 Florida Museum of Natural History, University of Florida, Gainesville, FL 32611, USA; 12 Department of Biology, University of Florida, Gainesville, FL 32611, USA; 13 New York Botanical Garden, Bronx, New York, NY 10458, USA; 14 Genome Research Center, Cold Spring Harbor Laboratory, 500 Sunnyside Blvd, Woodbury, NY 11797, USA; 15 Division of Biological Sciences, University of Missouri, Columbia, MI 65211, USA; 16 Departments of Biological Sciences and Medicine, Department of Biological Sciences, University of Alberta, Edmonton AB, T6G 2E9, Canada

## Abstract

**Background:**

Although it is agreed that a major polyploidy event, gamma, occurred within the eudicots, the phylogenetic placement of the event remains unclear.

**Results:**

To determine when this polyploidization occurred relative to speciation events in angiosperm history, we employed a phylogenomic approach to investigate the timing of gene set duplications located on syntenic gamma blocks. We populated 769 putative gene families with large sets of homologs obtained from public transcriptomes of basal angiosperms, magnoliids, asterids, and more than 91.8 gigabases of new next-generation transcriptome sequences of non-grass monocots and basal eudicots. The overwhelming majority (95%) of well-resolved gamma duplications was placed before the separation of rosids and asterids and after the split of monocots and eudicots, providing strong evidence that the gamma polyploidy event occurred early in eudicot evolution. Further, the majority of gene duplications was placed after the divergence of the Ranunculales and core eudicots, indicating that the gamma appears to be restricted to core eudicots. Molecular dating estimates indicate that the duplication events were intensely concentrated around 117 million years ago.

**Conclusions:**

The rapid radiation of core eudicot lineages that gave rise to nearly 75% of angiosperm species appears to have occurred coincidentally or shortly following the gamma triplication event. Reconciliation of gene trees with a species phylogeny can elucidate the timing of major events in genome evolution, even when genome sequences are only available for a subset of species represented in the gene trees. Comprehensive transcriptome datasets are valuable complements to genome sequences for high-resolution phylogenomic analysis.

## Background

Gene duplication provides the raw genetic material for the evolution of functional novelty and is considered to be a driving force in evolution [1,2]. A major source of gene duplication is whole genome duplication (WGD; polyploidy), which involves the doubling of the entire genome. WGD has played a major role in the evolution of most eukaryotes, including ciliates [3], fungi [4], flowering plants [5-16], and vertebrates [17-19]. Studies in these lineages support an association between WGD and gene duplications [6,20], functional divergence in duplicate gene pairs [21,22], phenotypic novelty [23], and possible increases in species diversity [24,25] driven by variation in gene loss and retention among diverging polyploidy sub-populations [26-29].

There is growing consensus that one or more rounds of WGD played a major role early in the evolution of flowering plants [2,5,7-9,13,30,31]. Early synteny-based and phylogenomic analyses of the *Arabidopsis *genome revealed multiple WGD events [8,9]. The oldest of these WGD events was placed before the monocot-eudicot divergence, a second WGD was hypothesized to be shared among most, if not all, eudicots, and a more recent WGD was inferred to have occurred before diversification of the Brassicales [9]. Synteny analyses of the recently sequenced nuclear genomes of *Vitis vinifera *(wine grape, grapevine) [32] and *Carica papaya *(papaya tree) [7] provided more conclusive evidence for a somewhat different scenario in terms of the number and timing of WGDs early in the history of angiosperms. Each *Vitis *(or *Carica*) genome segment can be syntenic with up to four segments in the *Arabidopsis *genome, implicating two WGDs in the *Arabidopsis *lineage after separation from the *Vitis *(or *Carica*) lineage [7,12,32]. The more ancient one (β) appears to have occurred around the time of the Cretaceous-Tertiary extinction [10]. Analyses of the genome structure of *Vitis *revealed triplicate sets of syntenic gene blocks [11,32]. Because the blocks are all similarly diverged, and thus were probably generated at around the same time in the past, the triplicated genome structure is likely to have been generated by an ancient hexaploidy event, possibly similar to the two successive WGDs likely to have produced *Triticum aestivum *[33]. Although the mechanism is not clear at this point, the origin of this triplicated genome structure is commonly referred to as gamma or γ (hereafter γ refers to the gamma event). Comparisons of available genome sequences for other core rosid species (including *Carica, Populus*, and *Arabidopsis*) and the recently sequenced potato genome (an asterid, *Solanum tuberosum*) show evidence of one or more rounds of polyploidy with the most ancient event within each genome represented by triplicated gene blocks showing interspecific synteny with triplicated blocks in the *Vitis *genome [7,11,34,35]. The most parsimonious explanation of these patterns is that γ occurred in a common ancestor of rosids and asterids, because all sequenced genomes within these lineages share a triplicate genome structure [12,35].

Despite this growing body of evidence from genome sequences, the phylogenetic placement of γ on the angiosperm tree of life remains equivocal (for example, [13]). As described above, the γ event is readily apparent in analyses of sequenced core eudicot genomes, and recent comparisons of regions of the *Amborella *genome and the *Vitis *synteny blocks indicate that the γ event occurred after the origin and early diversification of angiosperms [36]. In addition, comparisons of the *Vitis *synteny blocks with bacterial artificial chromosome sequences from the *Musa *(a monocot) genome provide weak evidence that γ postdates the divergence of monocots and eudicots [11].

As an alternative to synteny comparisons, a phylogenomic approach has also been used successfully to determine the relative timing of WGD events. By mapping paralogs created by a given WGD onto phylogenetic trees, we can determine whether the paralogs resulted from a duplication event before or after a given branching event [9]. In a recent study, Jiao *et al. *[5] used a similar strategy to identify two bouts of concerted gene duplications that are hypothesized to be derived from successive genome duplications in common ancestors of living seed plants and angiosperms. When using a phylogenomic approach, extensive rate variation among species could lead to incorrect phylogenetic inferences and then possibly also result in the incorrect placement of duplication events [11]. Gene or taxon sampling can reduce variation in branch lengths and the impact of long-branch attraction in gene tree estimates (for example, [37-39]). Therefore, effective use of the phylogenomic approach requires consideration of possible differences in substitution rates and careful taxon sampling to divide long branches that can lead to artifacts in phylogenetic analyses.

The availability of transcriptome data produced by both traditional (Sanger) and next-generation cDNA sequencing methods has grown rapidly in recent years [40,41]. In PlantGDB, very large Sanger EST datasets from multiple members of Asteraceae (for example, *Helianthus annuus*, sunflower) and Solanaceae (for example, *S. tuberosum*, potato), in particular, provide good coverage of the gene sets from the two largest asterid lineages. With advances in next-generation sequencing, comprehensive transcriptome datasets are being generated for an expanding number of species. For example, the Ancestral Angiosperm Genome Project has generated large, multi-tissue cDNA datasets of magnoliids and other basal angiosperms, including *Aristolochia, Persea, Liriodendron, Nuphar *and *Amborella *[5]. The Monocot Tree of Life project [42] is generating deep transcriptome datasets for at least 50 monocot species that previously have not been the focus of genome-scale sequencing. The 1000 Green Plant Transcriptome Project [43] is generating at least 3 Gb of Illumina paired-end RNAseq data from each of 1,000 plant species from green algae through angiosperms (Viridiplantae). In this study, we draw upon these resources, including an initial collection of basal eudicot species that have been very deeply sequenced by the 1000 Green Plant Transcriptome Project. Six members of Papaveraceae (*Argemone mexicana, Eschscholzia californica*, and four species of *Papaver*) have been targeted for especially deep sequencing, with over 12 Gb of cDNA sequence derived from four or five tissue-specific RNAseq libraries. Three other basal eudicots (*Podophyllum peltatum *(Berberidaceae), *Akebia trifoliata *(Lardizabalaceae), and *Platanus occidentalis *(Platanaceae)) sequenced by the 1000 Green Plant (1KP) Transcriptome Project, and EST sets available for additional strategically placed species (for example, [44,45]) were employed for phylogenomic estimation of the timing of the γ event. Assembled unigenes (sequences produced from assembly of EST data sets) were sorted into gene families and then the phylogenetic analyses of gene families were performed to test alternative hypotheses for the phylogenetic placement of the γ event.

## Results and discussion

**Figure 1:**
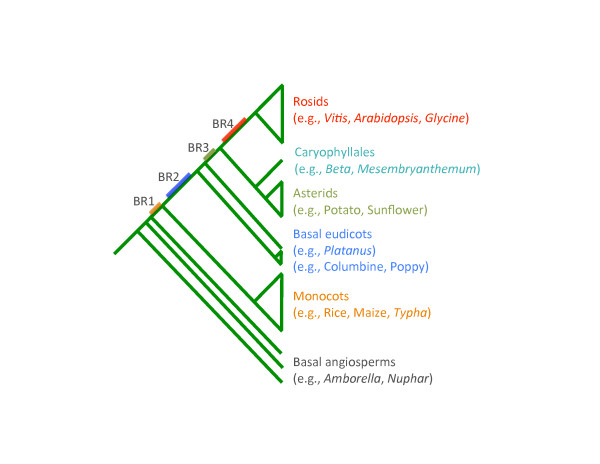
**Schematic phylogenetic tree of flowering plants**. BR1 to BR4 denote potential time points when the γ event may have occurred. BR1, monocots + eudicots duplication; BR2, eudicot-wide duplication; BR3, core eudicot-wide duplication; BR4, rosid-wide duplication.

Since the γ event was first identified in a groundbreaking phylogenomic analysis of the *Arabidopsis *genome [9], its timing has been hypothesized to have predated the origin of angiosperms (for example, [25,46]), the divergence of monocots and eudicots (for example, [47]) and the divergence of asterid and rosid eudicot clades (for example, [11,35]) (Figure 1). Most recent analyses suggest that γ occurred within the eudicots, but the timing of the γ event relative to the diversification of core eudicots remains unclear [13]. Resolving whether γ occurred just before the radiation of core eudicots or earlier, in a common ancestor of all eudicots, has implications for our understanding of the relationship between polyploidization, diversification rates, and morphological novelty (for example, [14]).

### Phylogenomic placement of the γ polyploidy event

To ascertain the timing of the γ event relative to the origin and early diversification of eudicots, we mainly focused on dating paralogous gene pairs that are retained on synteny blocks in *Vitis *[11,12]. *Vitis *displays the most complete retention for γ blocks among all genomes sequenced to date, and thus provides the best target for phylogenomic mining of the γ history. *Vitis *also represents the sister group to all other members of the rosid lineage (APG III, 2009) [48,49], so homologous genes were sampled from other species of rosids, asterids, basal eudicots, monocots, and basal angiosperms in order to estimate the timing of the γ event in relation to the divergence of these lineages. Genes were clustered into 'orthogroups' (homologous genes that derive from a single gene in the common ancestor of the focal taxa) using OrthoMCL [50] with eight sequenced angiosperm genomes (Table 1). By excluding *Vitis *pairs that are not included in the same orthogroups, and requiring that orthogroups contained both monocots and non-*Vitis *eudicots, 900 pairs of *Vitis *genes were retained from 781 orthogroups. These orthogroups were used in our investigation of the γ duplication event.

**Table 1:** Summary of datasets for eight sequenced plant genomes included in this study

Species	Annotation version	Number of annotated genes
*Arabidopsis thaliana *(thale cress)	TAIR version 9	27,379
*Carica papaya *(papaya)	ASGPB release	25,536
*Cucumis sativus *(cucumber)	BGI release	21,635
*Populus trichocarpa *(black cottonwood)	JGI version 2.0	41,377
*Glycine max *(soybean)	Phytozome version 1.0	55,787
*Vitis vinifera *(grape vine)	Genoscope release	30,434
*Oryza sativa *(rice)	RGAP release 6.1	56,979
*Sorghum bicolor*	JGI version 1.4	34,496

These eight genome sequences were used to construct orthogroups, which were then populated with additional unigenes of asterids, basal eudicots, non-grass monocots, and basal angiosperms. The number of annotated genes in each genome is indicated. ASGPB, Advanced Studies of Genomics, Proteomics and Bioinformatics; JGI, Joint Genome Institute; RGAP, Rice Genome Annotation Project; TAIR, The Arabidopsis Information Resource.

To verify that the phylogenetic placement of the γ event was shared by rosids and asterids, and to test whether it was shared by all eudicots or by eudicots and monocots (near angiosperm-wide), these orthogroups were then populated with unigenes of asterids, basal eudicots, non-grass monocots, and basal angiosperms (Table 2). Grasses are known to be distinct from other angiosperms in their high rate of nucleotide substitutions, and codon biases within the grasses make this clade distinct from other angiosperms, including non-grass monocots (for example, [51,52]), so inclusion of non-grass monocots was necessary to reduce artifacts in gene tree estimation. More generally, when dealing with phylogenomic-scale datasets, we strive for adequate taxon sampling to cut long branches, but avoid adding a large proportion of unigenes with low coverage. Inadequate taxon sampling could lead to spurious inference of phylogeny, while incomplete sequences (that is, low-coverage unigenes) can greatly degrade branch support and resolution of phylogenetic trees.

**Table 2:** Summary of unigene sequences of asterids, basal eudicots, non-grass monocots, and basal angiosperms included in phylogenetic study

Species	Lineage	Source	Number of reads/ESTs	Size of data	Assembly method(s)	Number of unigenes
*Panax quinquefolius*	Asterid	NCBI-SRA	209,745	89.7 Mb	MIRA	22,881
*Lindenbergia phillipensis*	Asterid	PPGP	69,545,362	5.9 Gb	CLC	104,904
*Helianthus annuus*	Asterid	TIGR PTA	93,279	NA	Megablast-CAP3	44,662
*Solanum tuberosum*	Asterid	TIGR PTA	219,485	NA	Megablast-CAP3	81,072
*Mimulus gutatus*	Asterid	PlantGDB	231,012	NA	Vmatch-PaCE-CAP3	39,577
*Papaver somniferum*	Basal eudicot	1KP + SRA	140,604,904 + 3,709,876	10.3 Gb + 1.3 Gb	MIRA-SOAPDenovo-CAP3	252,894
*Papaver setigerum*	Basal eudicot	1KP	134,478,938	9.8 Gb	SOAPDenovo-CAP3	406,167
*Papaver rhoeas*	Basal eudicot	1KP	157,506,374	11.5 Gb	SOAPDenovo-CAP3	383,426
*Papaver bracteatum*	Basal eudicot	1KP	89,663,900	6.5 Gb	SOAPDenovo-CAP3	201,564
*Eschscholzia californica*	Basal eudicot	NCBI + SRA + 1KP	14,381 + 559,470 + 133,422,402	6.8 Mb + 55 Mb + 9.7 Gb	MIRA-SOAPDenovo-CAP3	165,260
*Argemone mexicana*	Basal eudicot	1KP + NCBI	144,520,360 + 1,692	10.5 Gb + 1 Mb	SOAPDenovo- CAP3	148,533
*Akebia trifoliata*	Basal eudicot	1KP	29,156,514	2.1 Gb	CLC-CAP3	46,024
*Podophyllum pelatum*	Basal eudicot	1KP	20,139,210	1.5 Gb	CLC-CAP3	31,472
*Platanus occidentalis*	Basal eudicot	1KP	25,508,642	1.9 Gb	CLC-CAP3	42,373
*Aquilegia formosa *x *Aquilegia pubescens*	Basal eudicot	PlantGDB	85,040	NA	Vmatch-PaCE-CAP3	19,615
*Mesembryanthemum crystallinum*	Caryophillid	PlantGDB	27,553	NA	Vmatch-PaCE-CAP3	11,317
*Beta vulgaris*	Caryophillid	PlantGDB	25,883	NA	Vmatch-PaCE-CAP3	18,009
*Acorus americanus*	Monocot	MonATOL + 1KP	149,320 + 15,427,316	44.9 Mb + 1.1 Gb	MIRA-SOAPDenovo-CAP3	59,453
*Chamaedorea seifrizii*	Monocot	MonATOL	33,100,948	2.5 Gb	CLC	68,489
*Chlorophytum rhizopendulum*	Monocot	MonATOL	59,505,714	4.5 Gb	CLC	58,766
*Neoregelia *sp.	Monocot	MonATOL	49,121,506	3.7 Gb	CLC	63,269
*Typha angustifolia*	Monocot	MonATOL	70,733,124	5.7 Gb	CLC	57,980
*Persea americana *(avocado)	Magnoliid	AAGP	2,336,819	683 Mb	MIRA	132,532
*Aristolochia fimbriata *(Dutchman's pipe)	Magnoliid	AAGP	3,930,505	880 Mb	MIRA	155,371
*Liriodendron tulipifera *(yellow-poplar)	Magnoliid	AAGP	2,327,654	543 Mb	MIRA	137,923
*Nuphar advena *(yellow pond lily)	Basal angiosperm	AAGP	3,889,719	1.1 Gb	MIRA	289,773
*Amborella trichopoda*	Basal angiosperm	AAGP	2,943,273	776 Mb	MIRA	208394

1KP, 1000 Green Plant Transcriptome Project; AAGP, Ancestral Angiosperm Genome Project [44]; MonATOL, Monocot Tree of Life Project [42]; NA, not available; NCBI, National Center for Biotechnology Information; PPGP, Parasitic Plant Genome Project [65]; SRA, Sequence Read Archive; TIGR PTA, The Institute for Genomic Research Plant Transcript Assemblies [66].

**Figure 2:**
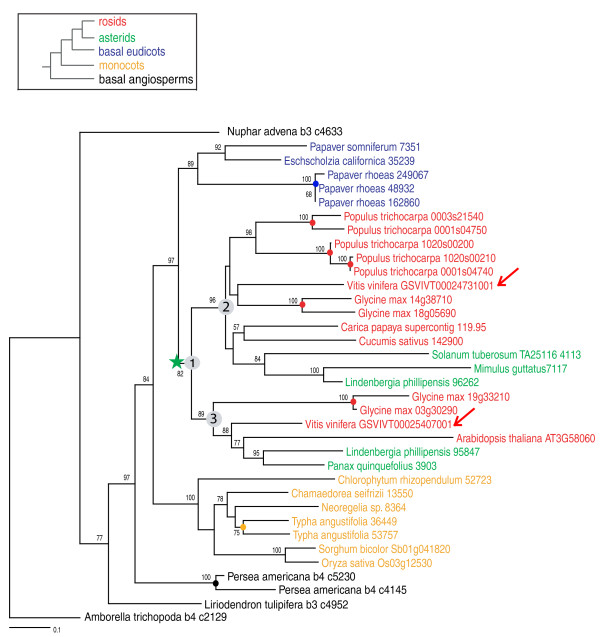
**Exemplar maximum likelihood phylogeny of Ortho 1202**. RAxML topology of an orthogroup (Ortho 1202) indicating that the γ paralogs of *Vitis *were duplicated before the split of rosids and asterids and after the early radiation of eudicots. The scored bootstrap (BS) value for this duplication is over 80%, because nodes #1 and #2 (and/or #3) have BS > 80%. Legend: green star = core eudicot duplication; colored circles = recent independent duplications; numbers = bootstrap support values.

**Figure 3:**
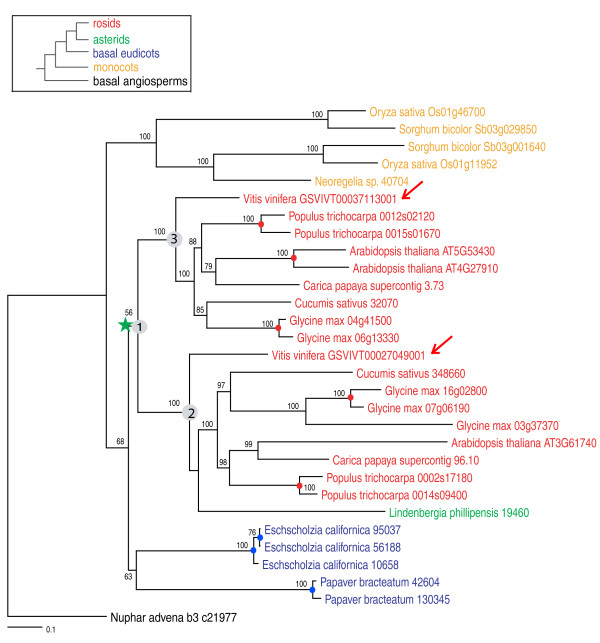
**Exemplar maximum likelihood phylogeny of Ortho 1083**. RAxML topology of an orthogroup (Ortho 1083) indicates that the γ paralogs of *Vitis *were duplicated before the split of rosids and asterids, and after the early radiation of eudicots. The scored bootstrap (BS) value for this duplication is over 50%, because nodes #1 has BS < 80%. Legend: green star = core eudicot duplication; colored circles = recent independent duplications; numbers = bootstrap support values.

To phylogenetically place the γ event with confidence, we adopted the following support-based approach. Three relevant bootstrap values were taken into account when evaluating support for a particular duplication. For example, given a topology of (((clade2)bootstrap2,(clade3)bootstrap3)bootstrap1), bootstrap2 and bootstrap3 are the bootstrap values supporting clade2 (clade2 here will include one of the *Vitis *γ duplicates) and clade3 (including the other *Vitis *duplicate), respectively, while bootstrap1 is the bootstrap value supporting the larger clade including clade2 and clade3. The value of bootstrap1 indicates the degree of confidence in the inferred ancestral node joining clades 2 and 3. In this study, when bootstrap1, and at least one of bootstrap2 and bootstrap3 were ≥50% (or 80%), we determined whether an asterid, basal eudicot, monocot, or basal angiosperm was contained in clades 2 or 3 (for example, asterids in Figures 2 and 3) or sister to their common ancestor (node defining clade 1) with a bootstrap value (BS) ≥50% (or 80%; for example, basal eudicots, monocots and basal angiosperms in Figures 2 and 3).

Homologous sequences were identified for 769 of the 781 orthogroups and were subsequently used for phylogenetic analysis. For example, orthogroup 1202 was well populated with unigenes of asterids, basal eudicots, non-grass monocots, and basal angiosperms (Figure 2). Two *Vitis *genes, which were located on a syntenic block, were clustered into two clades, both of which include genes from asterids and other rosids. This phylogenetic tree supports (BS ≥80%) the duplication of two *Vitis *genes before the split of rosids and asterids and after the divergence of basal eudicots, indicating that γ is restricted to core eudicots (BR3 of Figure 1; Figure 2). In another example, only one asterid unigene passed the quality control steps and was clustered into orthogroup 1083. This asterid unigene was grouped into one of the duplicated clades, also supporting (BS ≥50%) a duplication in the common ancestor of extant core eudicots (BR3 of Figure 1; Figure 3). Only a few duplications of *Vitis *gene pairs were identified as occurring before the divergence of monocots and eudicots (BR1 of Figure 1; seven duplications with BS ≥50%), or restricted to rosids (BR4 of Figure 1; six duplications with BS ≥50%, four duplications with BS ≥80%). We identified 168 *Vitis *gene pairs that were duplicated after the split of basal eudicots (BR3 of Figure 1) with BS ≥50%, and 80 of these had BS ≥80%. We also found that 70 *Vitis *genes were duplicated before the separation of basal eudicots (BR2 of Figure 1) with BS ≥50% and 19 with BS ≥80% (Table 3). Therefore, our phylogenomic analysis provided very strong support that γ occurred before the divergence of rosids and asterids, after the split of monocots and eudicots, and most likely after the earliest diversification of eudicots.

**Table 3:** Phylogenetic timing of *Vitis *γ duplications inferred from orthogroup phylogenetic histories

	BR1		BR2		BR3		BR4	
				
Ortho	BS ≥ 80	BS ≥ 50	BS ≥ 80	BS ≥ 50	BS ≥ 80	BS ≥ 50	BS ≥ 80	BS ≥ 50
Duplications	0	7	19	70	80	168	4	6
Percent	0%	2.8%	18.3%	27.9%	77.7%	67%	4%	2.3%

BRx designations are illustrated in Figure 1. Bootstrap (BS) ≥80 and BS ≥50 are counts of nodes resolved with BS ≥80 or ≥50, respectively.

### Molecular dating of the γ duplications

**Figure 4:**
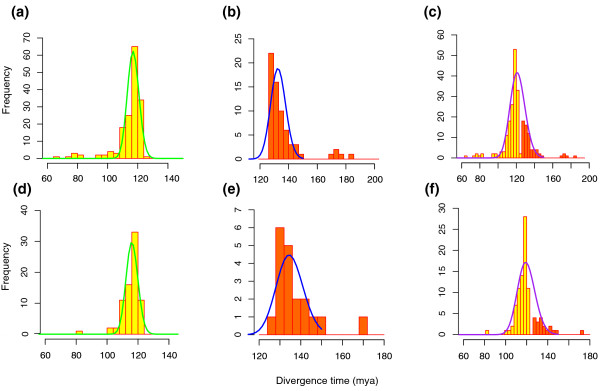
**Age distribution of γ duplications**. **(a) **The inferred duplication times for 161 γ duplication nodes that support core eudicot-wide duplication (BS ≥50%) were analyzed by EMMIX to determine whether these duplications occurred randomly over time or within some small timeframe. Each component is written as 'color/mean molecular timing/proportion' where 'color' is the component (curve) color and 'proportion' is the percentage of duplication nodes assigned to the identified component. There is one statistically significant component: green/117 (mya)/1. **(b) **Distribution of inferred γ duplication times from 66 orthogroups that support a eudicot-wide duplication with BS ≥50%. There is one statistically significant component: blue/133 (mya)/1. **(c) **Distribution of inferred γ duplication times from combination of (a) and (b) shows one significant component: purple/121 (mya)/1. **(d-f) **Corresponding distributions of inferred duplication times from orthogroups with BS ≥80%. One significant component in (d), green/116 (mya)/1; one in (e), blue/135 (mya)/1; and one in (f), purple/120 (mya)/1.

To estimate the absolute date of the γ event, we calibrated 161 of the 168 orthogroups supporting (BS ≥50%) a core eudicot-wide duplication and 66 of the 70 orthogroups supporting a eudicot-wide duplication, and then estimated the duplication times using the program r8s [53] (Materials and methods). We then analyzed the distribution of the inferred duplication times using a Bayesian method that assigned divergence time estimates to classes specified by a mixture model [54]. The distribution of duplication times of core eudicot-wide *Vitis *pairs shows a peak at 117 ± 1 (95% confidence interval) (Figure 4a), and the distribution of all eudicot-wide duplication times has a peak at 133 ± 1 million years ago (mya) (Figure 4b). Dating estimates have additional sources of error beyond the sampling effects accounted for in standard error estimates (for example, [55]). However, the clear pattern is that the duplication branch points occurred over a narrow window of time very close to the eudicot calibration point that represents the first documented appearance of tricolpate pollen in the fossil record. We also analyzed the 80 nodes and 19 nodes showing duplication shared by core eudicots and all eudicots, respectively, with bootstrap support ≥80% (Figure 4d, e) and found similar distributions (116 ± 1 mya for core eudicot duplications and 135 ± 2 mya for all eudicot duplications). The inferred dates for *Vitis *duplications shared either by core eudicots or all eudicots are very close to each other, and are concentrated around 125 mya. We also investigated the distribution of all inferred duplication times together (core eudicot-wide and eudicot-wide). Even given a time constraint (125 mya) that would split the date estimates for core eudicot and eudicot-wide duplications, the distributions of combined inferred duplication times show only one significant peak, with a mean at 121 mya for orthogroups with bootstrap support ≥50% (Figure 4c) and 120 mya for orthogroups with bootstrap support ≥80% (Figure 4f). A single peak observed for the combined data (Figure 4c) suggests that the genome-scale event(s) leading to the triplicated genome structure of core eudicots occurred in a narrow window of time nearly coincident with the sudden appearance of eudicot pollen-types in the fossil record [56].

### Hexaploidization and early eudicot radiation are close in time

Many of the gene trees showed no resolution or low bootstrap support for nodes distinguishing hypotheses BR2 and BR3. If the γ event had occurred almost anywhere along the long branch leading to eudicots, this event would have been relatively easy to resolve. The lack of resolution of the timing of duplication events around the basal eudicot speciation nodes suggests that the γ event may have occurred during a rapid species radiation. Another possibility could be due to the nature of hexaploidization. If, as our analyses suggest, the polyploidy event (see below for possible scenarios) occurred soon after the divergence of basal eudicots, the substitution rates for γ paralogs could vary. For example, one duplicate could evolve very slowly while the other evolves at an accelerated rate [4]. These possibilities could add significant challenges to the precise resolution of events occurring at or near the branch points for basal versus core eudicot lineages. Despite these challenges, most well-resolved gene trees support the hypothesis that the γ event occurred in association with the origin and diversification of the core eudicots, after the core eudicot lineage diverged from the Ranunculales (BR3 of Figure 1).

### Nature of the γ event

An additional question is whether the ancient hexaploid common ancestor was formed by one or two WGDs that occurred over a very short period (for example, as with hexaploid wheat). It was demonstrated that two of the three homologous regions were more fractionated than the third, suggesting a possible mechanism for the γ event [34]. In one proposed scenario, a genome duplication event generated a tetraploid, which then hybridized with a diploid to generate a (probably sterile) triploid. Finally, a second WGD event doubled the triploid genome to generate a fertile hexaploid. Alternatively, unreduced gametes of a tetraploid and a diploid could have fused to generate a hexaploid directly. Another characterization of syntenic blocks indicates that the three corresponding regions are generally equidistant from one another [11]. Our analyses of duplication points in the phylogenomic analyses resolve only a single peak in estimated dates for the 'γ event', which would be consistent with either scenario, given that any complex scenario would involve ancient events that occurred within a brief period of time. More evidence is needed to establish a more definitive mechanism for the apparent hexaploidization (that is, as one versus two events, allopolyploid versus autopolyploid).

### Rate variations between paralogs of *Vitis*

**Figure 5:**
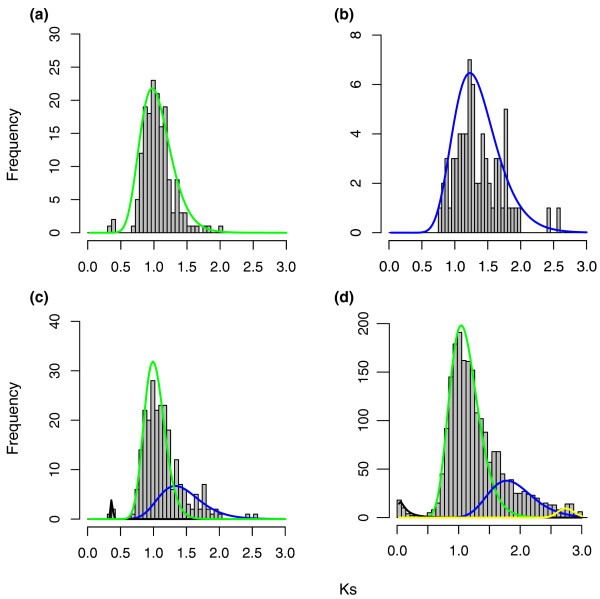
***K_s _*distributions of paralogs in *Vitis *from syntenic block analysis**. Methods for sequence alignment and estimation of *K_s _*were as reported (Cui *et al. *2006), but were here limited to paralogous gene pairs retained on syntenic blocks in the *Vitis *genome. Colored lines superimposed on *K_s _*distribution represent significant duplication components identified by likelihood mixture model as in Figure 4 (Materials and methods). a, *K_s _*distribution of 168 *Vitis *pairs supporting core eudicot-wide duplication in phylogenetic analysis. One statistically significant component: green/1.03/1. b, *K_s _*distribution of 70 *Vitis *pairs showing all eudicot-wide duplications on phylogenies. One significant component: blue/1.31/1. c, *K_s _*distribution of combination of *Vitis *pairs supporting core eudicot- (a) and eudicot-wide duplications (b) on phylogenies. Three significant components: black/0.3/0.01, green/1.02/0.70, blue/1.40/0.29. d, *K_s _*distribution of 2191 paralogous pairs were identified from syntenic block analysis. Four significant components: black/0.12/0.02, green/1.09/0.74, blue/1.85/0.22, yellow/2.7/0.02.

In another attempt to increase resolving power, *K_s _*distributions for duplicate *Vitis *genes were investigated. The *K_s _*distributions of *Vitis *pairs supporting a core eudicot-wide duplication inferred from phylogenetic analyses show one significant peak at *K_s _*~1.03 (Figure 5a). The *K_s _*values for eudicot-wide duplicate *Vitis *pairs were not well clustered, and their distribution shows one peak at 1.31, which indicates slightly more divergence for these *Vitis *pairs (Figure 5b). This result is consistent with phylogenetic analyses that show this set of duplications occurred somewhat earlier (all eudicot-wide versus core eudicot-wide). We also investigated the distribution of all *K_s _*values together (core eudicot-wide and eudicot-wide). Three statistically significant peaks were identified: 0.3, 1.02 and 1.40 (Figure 5c). Finally, we estimated *K_s _*values for all (2,191) pairs of *Vitis *γ paralogs identified by Tang *et al. *[11] in analyses of syntenic blocks. We were able to detect four significant components using the mixture model implemented with EMMIX (McLachlan *et al. *[54]): 0.12, 1.09, 1.85, and 2.7 (Figure 5d). This *K_s _*distribution clearly shows that the major peak (approximately 1.09; green curve in Figure 5d) was close to the peak of *K_s _*distribution of core eudicot-wide duplicates (at approximately 1.03; Figure 5a). This intriguing pattern (Figure 5c, d) could be a consequence of stable hexaploidy arising from two WGDs, one in the common ancestor of all eudicots and one in the common ancestor of core eudicots. However, there are no consistent patterns of duplications for entire syntenic blocks; for example, some syntenic blocks have genes consistently duplicated in core eudicots, while other syntenic blocks were duplicated eudicot-wide (results not shown). Alternatively, this pattern also could be consistent with the hypothesis of an allopolyploidy event for γ. If two ancestral genomes were involved in the hexaploidization and the *Vitis *genome had evolved slowly, two significant peaks might be detected [57]. A third possibility is that *Vitis *pairs supporting a eudicot-wide duplication may be the products of pre-WGD tandem or segmental duplications that were misidentified as syntenic γ paralogs due to loss of alternative copies through the fractionation process. These hypotheses will have to be tested through comparative analyses as additional plant genomes, especially of outgroups (for example, *Aquilegia, Amborella*) and other basal eudicots (eg., *Buxus, Trochodendron*), are sequenced.

### Implications of the γ event characterizing most eudicots

Our results suggest that the γ polyploidy event was closely coincident with a rapid radiation of major lineages of core eudicot lineages that together contain about 75% of living angiosperm species. This rapid lineage expansion following the γ event could be an important exception to the general pattern described by Mayrose *et al. *[31], who concluded that there may generally be reduced survival of polyploid plant lineages. The eudicots consist of a graded series of generally small clades (often called early-diverging or basal eudicots) that are successive sisters to the core eudicots ([49] and references therein). It is within the core eudicot clade where most major lineages as well as the large majority of angiosperm species reside (for example, rosids, asterids, caryophyllids). Several key evolutionary events seem to correspond closely to the origin of the core eudicots, including the genome-wide event described here, the evolution of a pentamerous, highly synorganized flower with a well-differentiated perianth, and the production of ellagic and gallic acids [58]. Significantly, the duplication of several genes crucial to the establishment of floral organ identity also occurred near the origin of the core eudicots (*AP3, AP1, AG*, and *SEP *gene lineages) [46,59,60], suggesting that these duplications - possibly originating from the γ event - may also be involved in the 'new' floral morphology that emerged in this clade [61,62].

This study also helps to shed light on prior studies, where the potential timing of the γ event varied widely from possibly in an ancestor of all angiosperms [9] to perhaps as recent as only rosids [63]. A polyploid event has been detected that is angiosperm-wide, but this was an earlier event (ε, epsilon) [5]. Our results are consistent with a recent study that identified a signature of the γ event in the genome of the potato, an asterid [35]. The γ event was suggested to be absent from grass genomes in comparisons of *Vitis *and *Oryza *[32], but this finding was questioned by Tang *et al. *[11]. However, the draft genome of strawberry (*Fragaria vesca*), a rosid that shares the γ event, did not show evidence for γ in syntenic block analysis [64], suggesting that either the γ event has been obscured by further rearrangements and fractionation, or expansion of the *Fragaria *genome sequence data may be necessary. Although sequenced plant genomes are being produced at an increasing rate, a much larger source of genome-scale evidence is coming from very large-scale transcriptome studies such as the 1000 Green Plant Transcriptome Project and the Monocot Tree of Life Project. In this paper, we have used gigabases of transcriptome data from species at key branch points to phylogenetically time hundreds of ancient gene duplications. Combined with evidence from *K_s _*analysis and syntenic blocks, global gene family phylogenies could incorporate extensive evidence without a sequenced genome, and ultimately facilitate a much better understanding of plant evolution.

## Conclusions

Phylogenetic analyses and molecular dating provide consistent and strong evidence supporting the occurrence of the γ polyploidy event after the divergence of monocots and eudicots, and before the asterid-rosid split. It is difficult to determine whether the γ event was shared by monocots or not based only on synteny patterns shared between *Vitis *and other monocot genomes [11]. By including massive transcriptome datasets from many additional taxa, such as basal angiosperms, non-grass monocots, basal eudicots and asterids, we employed a comprehensive phylogenomic approach, and dated gene pairs on syntenic blocks in a relatively slowly evolving species (*Vitis*) [11]. We were able to place the γ event(s) in a narrow window of time, most likely shortly before the origin and rapid radiation of core eudicots.

## Material and methods

### Data and assemblies

Genomes were obtained from various sources as given in Table 1. EST data or assemblies were obtained from sources indicated in Table 2. The largest quantities of new sequence data are represented by transcriptome datasets for nine basal eudicot species produced by Beijing Genomics Institute for the 1000 Green Plant Transcriptome Project [43]. The Monocot Tree of Life Project (MonATOL) generated five non-grass monocot transcriptomes. One transcriptome dataset for *Lindenbergia philippensis *(asterid) was obtained from the Parasitic Plant Genome Project [65]. Several methods were used for EST data assembly, according to the type and quantity of data that were available. Assemblies involving large numbers of Sanger reads were obtained either from the Plant Genome Database [45] or The Institute for Genomic Research (TIGR) Plant Transcript Assemblies [66]. Hybrid assemblies with Sanger and 454 data were performed with MIRA.Est. Short-read Illumina datasets were assembled either with *SOAP denovo *(K-mer size = 29 and asm_flag = 2) [67] or with CLC Genomics Workbench (reads trimmed first, and using default parameters except minimum contig length set to 200 bases). Assemblies for species with data from more than one sequencing technology were further post-assembled with CAP3 (overlap length cutoff = 40 and overlap percent identity = 98) to merge contigs that have significant overlap but could not be assembled into contiguous sequences by primary assemblers due to either the presence of SNPs in the consensus or path ambiguity in the graph.

### Gene classification and phylogenetic analysis

The OrthoMCL method [50] was used to construct sets of orthogroups. Amino acid alignments for each orthogroup were generated with MUSCLE, and then trimmed by removing poorly aligned regions with trimAl 1.2, using the heuristic automate1 option [68]. In order to sort and align transcriptome data into our eight-genome scaffold for downstream phylogenetic analyses, we first used ESTScan [69] to find the best reading frame for all unigenes. The best hit from a blast search against the inferred proteins of our eight-genome scaffold was then used to assign each unigene to an orthogroup. Additional sorted unigene sequences for the orthogroups of sequenced genomes were aligned at the amino acid level into the existing full alignments (before trimming) of eight sequenced species using ClustalX 1.8 [70]. Then these large alignments were trimmed again using trimAl 1.2 with the same settings. Each unigene sequence was checked and removed from the alignment if the sequence contained less than 70% of the total alignment length. Corresponding DNA sequences were then forced onto the amino acid alignments using custom Perl scripts, and DNA alignments were used in subsequent phylogenetic analysis. Maximum likelihood analyses were conducted using RAxML version 7.2.1 [71], searching for the best maximum likelihood tree with the GTRGAMMA model by conducting 100 bootstrap replicates, which represents an acceptable trade-off between speed and accuracy (RAxML 7.0.4 manual).

### Molecular dating analyses and 95% confidence intervals

The best maximum-likelihood topology for each orthogroup was used to estimate divergence times. The divergence time of the two paralogous clades in each orthogroup was estimated under the assumption of a relaxed molecular clock by applying a semi-parametric penalized likelihood approach using a truncated Newton optimization algorithm as implemented in the program R8S [53]. The smoothing parameter was determined by cross-validation. We used the following dates in our estimation procedure: minimum age of 131 mya [72] and maximum age of 309 mya for crown-group angiosperms [73], and a fixed constraint age of 125 mya for crown-group eudicots [56]. We required that trees pass both the cross-validation procedure and provide estimates of the age of the duplication node. The collection of inferred divergence times was then analyzed by EMMIX [54]. For each significant component identified by EMMIX, the 95% confidence interval of the mean was then calculated.

### Finite mixture models of genome duplications

To explore the divergence patterns for duplicated genes, the inferred distribution of *K_s _*divergences were fitted to a mixture model comprising several component distributions in various proportions. The *K_s _*value for each duplicated sequence pair was calculated using the Goldman and Yang maximum likelihood method implemented in codeml with the F3X4 model [74]. The EMMIX software was used to fit a mixture model of multivariate normal components to a given data set. The mixed populations were modelled with one to four components. The EM algorithm was repeated 100 times with random starting values, as well as 10 times with *k*-mean starting values. The best mixture model was identified using the Bayesian information criterion.

## Abbreviations

BS: bootstrap value; EST: expressed sequence tag; *K_s_*: rate of synonymous substitutions per synonymous site; mya: million years ago; WGD: whole genome duplication.

## Authors' contributions

YJ, JL-M and CWD conceived of the study and its design, and YJ performed all of the final analyses. YJ, JL-M, CWD drafted the primary manuscript and additional text and discussion of the research was provided by DES, PSS, JEB, NJW, TMK, GW, DWS. Tissue samples, RNA isolations, library preparation sequencing and sample and sequence management were done by MR, MRM, JM, MR, XW, YongZ, JW, ASC, MKD, RM and JCP. Data assemblies and other analyses were done by YJ, SA, DRR, EW, and YetingZ. All authors contributed to and approved the final manuscript for publication.
